# Correlation between parity and metabolic syndrome in Chinese women aged 40 years and older: the Reaction study

**DOI:** 10.1186/s12902-021-00902-7

**Published:** 2021-11-24

**Authors:** Qian Xie, Haoran Xu, Qin Wan

**Affiliations:** 1 Department of Gerontology, the people’s Hospital of LeShan, LeShan, 614000 China; 2 The first clinical institute, ZunYi Medical University, ZunYi, 5630066 China; 3 Department of Endocrinology, the Affiliated Hospital of Southwest Medical University, Luzhou, 641400 China

**Keywords:** Live-birth pregnancies, Metabolic syndrome, Menopause, Diabetes, Hypertension, Obesity

## Abstract

**Aims:**

The purpose of the present study was to investigate the correlation between the number of live-birth pregnancies and metabolic syndrome (MetS) in Chinese women according to menstruation history.

**Methods:**

Registry data for all pregnancies in a cohort of 6157 Chinese women aged 40 years or older were obtained and the number of live-birth pregnancies were enumerated. We defined MetS using five criteria: impaired insulin metabolism and glucose tolerance, obesity in the abdominal area, dyslipidemia, and hypertension. Multivariate logistic regression analysis was conducted to assess potential risk factors for MetS. Postmenopausal women with three or more of live-birth pregnancies had the highest prevalence of MetS (*P* < 0.05).

**Results:**

Among the 6157 females aged 40 years or older in Luzhou city, 2143 (34.8%) participants had incident MetS. The number of live-birth pregnancies was significantly correlated with age and fasting blood glucose (FBG) level (*P* < 0.05). The prevalence of MetS increased with the number of live-birth pregnancies (*P* < 0.01), and the frequency in postmenopausal women was significantly higher than that in premenopausal women (*P* < 0.001). In the binary logistic regression model, menopausal status [OR = 0.343 (0.153–0.769), *P* < 0.001] were significantly associated with an increased risk of MetS.

**Conclusions:**

The number of live-birth pregnancies is correlated with an increased risk of MetS in Chinese women aged 40 years and over, especially in postmenopausal women. Greater attention should be paid to postmenopausal women who have had multiple live-birth pregnancies with a view to intervening early to prevent related diseases.

## Introduction

Metabolic syndrome (MetS) is a multifaceted disease characterized by impaired insulin metabolism and glucose tolerance, obesity in the abdominal area, dyslipidemia, and hypertension [1]. According to statistics, approximately 20–25% of the population worldwide suffers from this syndrome [2]. The prevalence of MetS in the U.S. population has been estimated to be 25.0% [3], and a meta-analysis conducted in mainland China demonstrated a pooled frequency of 24.5% (19.25% in males and 27.0% in females) [4]. The incidence of MetS continues to increase, causing serious economic burden to society, especially in developing countries. Reports have indicated a high prevalence of MetS in the Philippines (19.7%) [5], Malaysia (27.5%) [6], Nigeria (28.1%) [7], India (28.2%) [8], Brazil (29.6%) [9], Iran (36.9%) [10], and Turkey (44.0%) [11]. MetS is considered to increase the risk of all-cause and cardiovascular disease (CVD) mortality, incident stroke, cancer, and sleep disorders [12–15]. A recent study showed that individuals with MetS have a 2-fold increased risk of developing CVD and a 5-fold increased risk of developing type 2 diabetes as compared with individuals without MetS [16].

Although pregnancy is a limited process, its impact on a woman’s physiological status is lengthy and marked; thus, middle-aged and elderly multiparous women are at a high risk of MetS. Pregnancy leads to significant metabolic changes, such as increased production of insulin; reduced insulin sensitivity; and increased fat mass, triglycerides, low-density lipoprotein cholesterol, and blood glucose [17, 18]. Although these changes that occur during pregnancy are almost completely reversed following delivery, the lasting effects of the state of inflammation can result in an increased risk of hypertension and CVD in later life [19,20]. A Danish cohort study found that the highest risk of developing hypertension and CVD in premenopausal women is during their first live-birth pregnancy and after their fourth [21]. A follow-up study in the U.S. indicated that BMI in women aged 45–74 is significantly positively correlated with the number of live-birth pregnancies [22]. A recent study showed that older multiparous women are more likely to develop impaired glucose regulation (IGR) and diabetes than their counterparts with fewer children. Moreover, it has been reported that the number of children is also associated with MetS and diabetes in men [23]. However, knowledge is lacking regarding the correlation between MetS and the number of live-birth pregnancies in Chinese women aged 40 years and over; thus, in the present study, we investigated this correlation, in addition to the association between menopause and MetS.

## Methods

### Study population

The cross-sectional study data were taken from REACTION [24], a longitudinal study investigating cancer risk in Chinese diabetics, which was performed between April and November 2011 by the Chinese Medical Association endocrine branch. In brief, the baseline data for 6157 females aged 40 years or older in Luzhou city were selected. Extensive training relative to the study questionnaire and outcome measures was received by the investigators prior to the beginning of the investigation. Each participant provided written informed consent relating to participation in this study. Following the ethical standards in Declaration of Helsinki, ethical approval of this study was obtained from the Research Ethics Committee, the Affiliated Hospital of Southwest Medical University. Inclusion criteria were: 1) permanent residents aged ≥40 years; 2) gender: female; and 3) good compliance. Subjects were excluded according to the following parameters: pregnant women, lack of mobility, old age(> 85-year-old), weakness, communication barriers, poor compliance, history of long-term chronic diseases, recent acute diseases, and women who had never given birth.

The National Cholesterol Education Program (NCEP ATP III) criteria [15] for MetS were used to classify subjects without previously known MetS when three of the following five components were present: 1) abdominal obesity: female waist circumference ≥ 85 cm; 2) increased blood glucose: fasting blood glucose ≥6.1 mmol/L, blood glucose ≥7.8 mmol/L 2 h after a meal and/or diagnosed with diabetes, currently under anti-diabetic treatment; 3) hypertension: blood pressure ≥ 130/85 mmHg and/or diagnosed with hypertension; 4) on an empty stomach HDL-C < 1.04 tendency/L; 5) fasting triglycerides (TG) ≥ 1.7 mmol/L.

### Other measures

During the baseline interview, all participants were asked about gender, age, family history of diabetes, degree of education, age at menopause, age at which their first child was born, alcohol consumption and smoking habits, and number of live-birth pregnancies (excluding other children: adopted, fostered, step). Smoking status was defined as current smoking and still smoking 1 year prior to baseline.

A physical examination was carried out, during which blood pressure was measured at the right brachial artery 3 times in a seated position, at 5-min intervals, using an automatic sphygmomanometer. Height and body weight were measured with the individual wearing light-weight clothes and no shoes, and body mass index (BMI) was subsequently calculated by dividing the weight (kg) by the height (m)^2^. Measurement of the waist circumference was performed at the midway level between the iliac crest and the costal margin.

Blood samples were taken following an 8-h overnight fast. All subjects underwent an OGTT (82.5 g glucose). The glucose oxidase test and colorimetric enzyme assays were employed to determine plasma levels of glucose, triglycerides (TG), hemoglobin A1c, high-density lipoprotein cholesterol (HDL-C), and low-density lipoprotein cholesterol (LDL-C). Informed consent was obtained from all subjects.

### Statistical analysis

The database was established using the Epidata software. Continuous variables are expressed as the mean ± SD, and two-sided *P* < 0.05 was considered statistically significant. Continuous variables were analyzed using one-way analysis of variance, If the difference was statistically significant, Bonferroni method was used for pairwise comparison. Categorical variables were analyzed via the χ2 test. Logistic regression was used to evaluate the influence of factors related to the number of live-birth pregnancies on MetS. SPSS 19.0 was used for statistical analysis.

## Results

A total of 6157 females aged 40 years and over were selected for the present study (average age, 58.7 ± 10.1 years). In China, women who have never given birth account for less than 1% of the total number of women over 40 and were therefore excluded. Women were grouped according to the number of live-birth pregnancies: women with 1 live birth, one (3754, 60.97%); women with 2 live births, two (1459, 23.70%); women with 3 live births, three (603, 9.79%); and women with 4 and more live births, four or more (341, 5.54%). The characteristics of each group are shown in Table 1. The results indicate that the number of live-birth pregnancies (*P* < 0.01) and FBG (*P* < 0.01) increased with age. The family history of diabetes was significantly lower as the number of live-birth pregnancies increased (*P* < 0.05). Women with 1 live birth and 2 live births had a lower 2hPG, HbA1c, waist circumference, and SBP as compared with those in the other two groups (*P* < 0.05). TG, BMI and hip circumference in women with 1 live birth were significantly lower than those in the other groups (*P* < 0.05), and HDL-C in women with 1 live birth was significantly higher than that in the other groups (*P* < 0.01). However, there was no significant difference in 2hPG, HbA1c, TC, TG, HDL-C, LDL-C, waist circumference, hip circumference, or SBP between women with 3 live births and 4 live births. The number of live-birth pregnancies did not cause a significant improvement in DBP (Table 1).

**Table 1:** Characteristics of the study participants according to the number of live-birth pregnancies

The number of live-birth pregnancies (number)						
Variable	1 (3754, 60.97%)	2 (1459,23.70%)	3 (603, 9.79%)	≥4 (341, 5.54%)	F or *χ*^2^	***P***
Age (years)	53.8 ± 7.9^▲□■^	60.3 ± 9.0^△□■^	67.7 ± 7.1^△▲■^	72.0 ± 6.4^△▲□^	1028.094	< 0.001
FBG (mmol/L)	5.73 ± 1.44^▲□■^	5.92 ± 1.60^△■^	6.08 ± 1.60^△■^	6.25 ± 1.77^△▲□^	24.524	< 0.001
2hPG (mmol/L)	7.24 (6.10,9.17)^▲□■^	8.24 (6.70,10.67)^△□■^	8.90 (7.29,12.37)^△▲^	9.24 (7.51,12.88)^△▲^	80.341	< 0.001
HbA1c (%)	5.99 ± 0.96^▲□■^	6.20 ± 1.04^△□■^	6.34 ± 1.08^△▲^	6.44 ± 1.29^△▲^	42.710	< 0.001
TC (mmol/L)	4.68 ± 1.14^□^	4.73 ± 1.20	4.85 ± 1.22^△^	4.77 ± 1.12	4.313	0.005
TG (mmol/L)	1.25 (0.89,1.80)^▲□■^	1.36 (0.96,2.01)^△^	1.42 (1.05,2.09)^△^	1.37 (0.97,1.94)^△^	58.816	< 0.001^a^
HDL-C (mmol/L)	1.31 ± 0.35^▲□■^	1.26 ± 0.34^△^	1.27 ± 0.33^△^	1.26 ± 0.32^△^	10.831	< 0.001
LDL-C (mmol/L)	2.61 ± 0.82^□^	2.65 ± 0.86	2.73 ± 0.88^△^	2.70 ± 0.85	4.688	0.003
BMI (kg/m^2^)	23.5 ± 3.2^▲□■^	24.3 ± 3.2^△^	24.3 ± 3.5^△^	24.2 ± 3.5^△^	24.088	< 0.001
Waist circumference (cm)	80.0 ± 9.8^▲□■^	83.2 ± 10.0^△□■^	84.8 ± 10.0^△▲^	85.3 ± 11.2^△▲^	79.751	< 0.001
Hip circumference (cm)	92.7 ± 9.2^▲□■^	94.4 ± 9.3^△^	95.0 ± 8.4^△^	95.1 ± 8.8^△^	22.727	< 0.001
SBP (mmHg)	120.0 ± 18.5^▲□■^	126.7 ± 19.4^△□■^	133.2 ± 21.3^△▲^	134.9 ± 19.2^△▲^	147.387	< 0.001
DBP (mmHg)	75.3 ± 10.4	76.0 ± 10.4	75.5 ± 11.1	74.6 ± 11.7	2.272	0.078
Family history of diabetes (%)	23.8%	14.3%	11.7%	7.5%	113.133	< 0.001^b^
Age when first gave birth birth	20.6 ± 4.4^■^	20.3 ± 4.8^■^	20.2 ± 5.2^■^	21.6 ± 6.7^△▲□^	2.130	0.094
Menopausal status (Yes/No)	2687/1067	1256/203	585/18	339/2	358.481	< 0.001^b^

Abbreviations: DBP, diastolic blood pressure; FBG, fasting blood glucose; 2hPG, postprandial blood glucose; SBP, systolic blood pressure, LDL-C, low-density lipoprotein cholesterol; HDL-C, high-density lipoprotein cholesterol

^△^*P* < 0.05 as compared with group 1; ^▲^*P* < 0.05 as compared with group 2; ^□^*P* < 0.05 as compared with group 3; ^■^*P* < 0.05 as compared with group 4

Among the 2143 (34.8%) participants with incident MetS, there were 1017 (27.1%) in women with 1 live birth, 618 (42.4%) in women with 2 live births, 325 (53.9%) in women with 3 live births, and 183 (53.7%) in women with 4 and more live births. The prevalence of MetS increased significantly as the number of live-birth pregnancies increased (*P* < 0.01), with women with 4 and more live births having the highest prevalence of MetS (*P* < 0.05) (Table 2).

**Table 2:** Comparison of the prevalence of MetS among the four groups

Number of live-birth pregnancies	MetS	Total	Morbidity (%)	*χ*^2^	***P***
1	1017	3754	27.1	229.962	< 0.001
2	618	1459	42.4		
3	325	603	53.9		
**≥4**	183	341	53.7		
**Total**	2143	6157	34.8		

Table 3 presents the distribution of MetS morbidity under different menstruation situations. The prevalence of MetS in women over 40 years old was 34.8%, of which premenopausal women accounted for 16.6% and postmenopausal women accounted for 39.2%. The significant difference was found between pre- and postmenopausal women (*P* < 0.001). In the women with 1 live birth, 2 live births and 3 live births, the frequency of MetS was significantly higher in postmenopausal women than that in premenopausal women (*P* < 0.001). The prevalence of MetS in premenopausal women was significantly increased as the number of live-birth pregnancies increased (*P* < 0.05). Postmenopausal women showed an increasing trend in the prevalence of MetS as the number of live-birth pregnancies increased (*P* < 0.001), with women in women with 4 and more live births having the highest prevalence of MetS (*P* < 0.05) (Table 3).

**Table 3:** The distribution of MetS morbidity under different menstruation situations

Number of live-birth pregnancies	Premenopausal			Postmenopausal			*χ*^2^	***P***
	MetS	Total	Morbidity %	MetS	Total	Morbidity %		
1	167	1067	15.7	846	2687	31.5	78.183	< 0.001
2	41	203	24.3	497	1099	45.2	26.350	< 0.001
3	5	20	25.0	320	583	54.9	4.142	0.042
**≥4**	2	2	100.0	181	339	53.4	0.862	0.353
**Total**	215	1292	16.6	1844	4708	39.2	62.457	< 0.001
*χ*^2^	9.542			135.877			–	–
***P***	0.002			< 0.001			–	–

In the binary logistic regression model, we found that FBG [OR = 1.629, *P* < 0.05], 2hPG [OR = 1.394, *P* < 0.001], BMI [OR = 1.113, *P* = 0.049], TG [OR = 4.682, *P* < 0.001], HDL-C [OR = 0.009, *P* < 0.001], SBP [OR = 1.055, *P* < 0.001], waist circumference [OR = 1.143, *P* < 0.001], and menopausal status [OR = 0.343, *P* < 0.001] were significantly associated with an increased risk of MetS, whereas elevated age, HbA1c, TC, pregnancy number, DBP, hip circumference, and age at which the first child was born were not. The analysis results indicate that the number of live-birth pregnancies [OR = 1.368, *P* = 0.154] was only a suspect factor that did not reach statistical significance. Moreover, FBG, 2hPG, TG, HDL-C, menopausal status, SBP, and waist circumference were all independent risk factors for MetS following adjustment for other factors (Table 4).

**Table 4:** Multiple logistic regression analysis of risk factors for MetS

Variable	***B***	***S.E***	***Wald***	***P***	***OR***	95%***CI***
**Number of live-birth pregnancies**	0.314	0.220	2.032	0.154	1.368	0.889 ~ 2.106
**Age**	0.043	0.023	3.673	0.055	1.044	0.999 ~ 1.092
**FBG**	0.464	0.150	9.574	0.002	1.629	1.468 ~ 1.843
**2hPG**	0.332	0.053	40.001	0.000	1.394	1.258 ~ 1.545
**BMI**	0.107	0.054	3.867	0.049	1.113	1.000 ~ 1.237
**TG**	1.544	0.314	24.122	0.000	4.682	2.529 ~ 8.869
**HDL-C**	−4.700	0.941	24.940	0.000	0.009	0.001 ~ 0.058
**Menopausal status**	−1.070	0.412	6.752	0.009	0.343	0.153 ~ 0.769
**SBP**	0.054	0.009	33.265	0.000	1.055	1.036 ~ 1.075
**Waist circumference**	0.133	0.022	35.599	0.000	1.143	1.094 ~ 1.194

OR was computed using the binary logistic regression model. All covariables were included simultaneously in the model. Elevated age, HbA1c, TC, number of live-birth pregnancies, DBP, hip circumference, and age at which the first child was born were not significantly associated with an increased risk of diabetes and were therefore not included in the final model

B, unstandardized regression coefficients; SE, standard error; OR, odds radio

## Discussion

MetS is a serious public health concern resulting from a sedentary lifestyle and poor diet. The prevalence of MetS has been shown to be significantly higher in women than that in men. In the present study, we investigated the correlation between the number of live-birth pregnancies and MetS in women aged 40 years and over in Sichuan, China. It has previously been reported that the pooled incidence of MetS in China is 33.9% (31.0% in men and 36.8% in women), indicating that approximately 454 million adults are affected [25]. At present, the pathogenesis of MetS remains unclear, and its occurrence is the result of a combination of factors including those of genetic and social environmental origin. One study found that MetS is linked to older age, lower educational level, and high levels of uric acid, alanine transaminase (ALT), gamma-glutamyl transferase (γ-GT), and creatinine in a Taiwanese cohort [26]. Goeun et al. [27] demonstrated that among men and women aged 50–64 years, living without a spouse, having a low educational level, and reporting a low economic status were associated with MetS prevalence. In a cross-sectional study of 1326 women, Goh et al. [28] observed that those with abdominal obesity are more likely to suffer from MetS, suggesting that waist circumference increases the risk of developing MetS. Moreover, pregnancy can lead to obesity due to the accumulation of body fat, reduction in physical activity, and increased calorie diet.

However, the notion that the number of live-birth pregnancies is an independent predictor of MetS remains controversial. In a cross-sectional study involving a total of 1251 elderly women (aged 60–95 years) [29], a strong association was observed between reproductive variables and a higher risk of MetS; women who had given birth a larger number of times had elevated ORs for MetS. When using the first tertile of the number of live-birth pregnancies as a reference, ORs for the second and third tertiles were 1.36 (95% CI: 0.95–1.96) and 1.75 (95% CI: 1.19–2.57) respectively, following adjustment for age, marital status, educational level, current smoking habit, current consumption of alcohol, 30 min of physical activity per day, BMI, and family history of CVD. A cross-sectional study of 4098 postmenopausal women performed using the Korean National Health and Nutrition Examination Survey found that the more times a woman gives birth, the higher the risk of MetS [30]. In contrast, another study found that increasing parity has no effect on insulin sensitivity or β-cell function [31]. A further study showed that multiple live-birth pregnancies are linked with the development of diabetes in elderly women, which appeared to be confounded and/or mediated by weight fluctuations and sociodemographic parameters. Higher parity does not appear to pose an ongoing risk of developing diabetes in older women [32]. Cohen et al. [33] observed that the rate of MetS among a national sample of women increased as the number of live-birth pregnancies increased; however, the strength of these correlations reduced following additional adjustment for BMI, which indicated that weight or changes thereof could be a vital mediator of the effect of the number of live-birth pregnancies on MetS risk. This is in accordance with the results of our study, in which the rate of participants with incident MetS was 34.8%. We found that groups 3 and 4 had an approximate 2-fold increased prevalence of MetS as compared with that in women with 1 live birth. The prevalence of MetS significantly increased as the number of live-birth pregnancies increased; however, it was not an independent risk factor for MetS following adjustment for age, FBG, 2hPG, BMI, TG, HDL-C, SBP, waist circumference, menopausal status, bA1c, TC, pregnancy number, DBP, hip circumference, and age. Increased risk of MetS could obscure the influencing power of parity.

In the present study, we also found that the prevalence of MetS in premenopausal women was 16.6% and that in postmenopausal women was 39.2%, which are similar results to those of a meta-analysis showing that the global prevalence rate of MetS in postmenopausal women is 37.17%, much higher than that in premenopausal women. A cross-sectional study [34] conducted in a sample of 640 women aged 40–65 years old showed that the prevalence of MetS varies with menopausal status: 45.7% in premenopausal women and 57.5% in postmenopausal women. A retrospective study in 958 women aged 40–65 years old conducted in southern Brazil found that the incidence of MetS in postmenopausal women (22.2%) was much higher than that in premenopausal women (8.4%) (RR = 2.75), suggesting that menopausal status affects the incidence of MetS in women [35]. These data are consistent with those from a Korean study showing an OR of 2.93 for the incidence of MetS in post- as compared with premenopausal women after controlling for age, BMI, and other confounders [25]. Another study showed that the longer a woman has been postmenopausal, the higher the occurrence of MetS, with the OR increasing from 1.40 to 1.58 in a time-dependent manner [29]. These observations are similar to those in our study showing that postmenopausal status [OR = 0.343 (0.153–0.769), *P* < 0.001] was an independent risk factor for MetS.

The present study shows that the incidence of MetS in postmenopausal women was significantly higher than that in premenopausal women (*P* < 0.001) when the number of live-birth pregnancies was less than 3. Postmenopausal women showed an increasing trend in the prevalence of MetS as the number of live-birth pregnancies increased (*P* < 0.001). Moreover, postmenopausal women with increasing numbers of live-birth pregnancies had an approximate 2-fold increased prevalence of MetS as compared with that of premenopausal women. Postmenopausal women who had had three or more live-birth pregnancies had the highest prevalence of MetS (*P* < 0.05). Previous studies have indicated that both hormone therapy and individualized lifestyle intervention can reduce the incidence of MetS in postmenopausal women [36]. Greater attention should be paid to postmenopausal women who have had a greater number of live-birth pregnancies with a view to preventing related chronic diseases.

The specific mechanism underlying the effect of the number of live-birth pregnancies and menopausal status on MetS remains unknown but may be related to the following three aspects. Firstly, pregnancy is a temporary, non-physiological condition for a menstrual woman such as insulin resistance, dyslipidemia, fat accumulation, inflammation, and weight gainbut most of these alterations revert to the nonpregnant state after delivery, some changes could persist and even accumulate across successive pregnancies [32]. Previous research has reported waist girth increases with each birth, while bodyweight increased only after the first birth which was consistent with our research [37]. Secondly, most mothers spend the majority of their time caring for children and change in lifestyle risk factors; therefore, reduced physical exercise can lead to obesity, further insulin resistance, and enhanced glucocorticoid activity [38]. Thirdly, the levels of sex hormones change in menopausal women and the estrogen level begins to decline. Since the protective effect of estrogen is weakened, disorders related to blood glucose and lipid metabolism can easily develop, which increase the risk of CVD, diabetes, and other metabolic disorders [39].

This study has limitations. The study only looks at chinese women. No information was available about the pregnancy state, such as gestational weight gain, postpartum weight retention or pregnancy complications, in the current study.

In summary, the number of live-birth pregnancies was associated with a higher risk of MetS. Postmenopausal women who have had three or more live-birth pregnancies had the highest prevalence of MetS; therefore, attention should be paid to these individuals with a view to preventing related chronic diseases.

## Conclusions

The number of live-birth pregnancies is correlated with an increased risk of MetS in Chinese women aged 40 years and over, especially in postmenopausal women. Greater attention should be paid to postmenopausal women who have had multiple live-birth pregnancies with a view to intervening early to prevent related diseases.

## Data Availability

The datasets used and/or analysed during the current study are available from the corresponding author on reasonable request.

## Abbreviations

MetS: metabolic syndrome
CVD: cardiovascular disease
IGR: impaired glucose regulation
TG: triglycerides
HDL-C: high-density lipoprotein cholesterol
LDL-C: low-density lipoprotein cholesterol
FBG: fasting blood glucose
2hPG: postprandial blood glucose
DBP: diastolic blood pressure
SBP: systolic blood pressure
BMI: body mass index
